# Supplementary material for: Free-space creation of ultralong anti-diffracting beam with multiple energy oscillations adjusted using optical pen
Source: Nat Commun. 2018 Nov 28;9:5035. doi: 10.1038/s41467-018-07282-y (PMC6262014; doi:10.1038/s41467-018-07282-y)
Supplement: Supplementary file 1 — Supplementary Information [file 41467_2018_7282_MOESM1_ESM.pdf]

Supplementary Information

Free-space creation of ultralong anti-diffracting beam with multiple energy oscillations adjusted using optical pen

Weng et al.

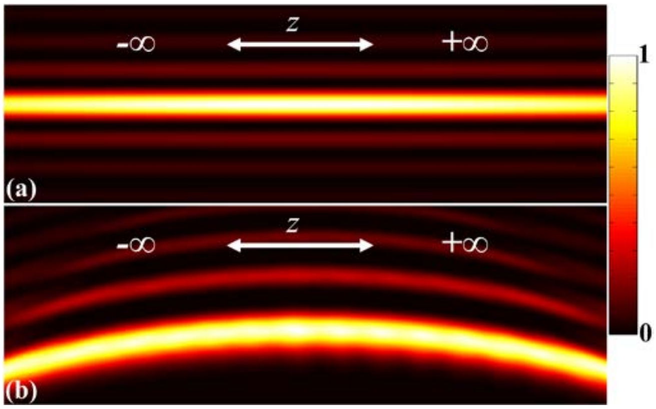

Supplementary Figure 1 Normalized light intensity of ideal anti-diffracting light beams along *z* axis. (a) Bessel beam; (b) Airy beam.

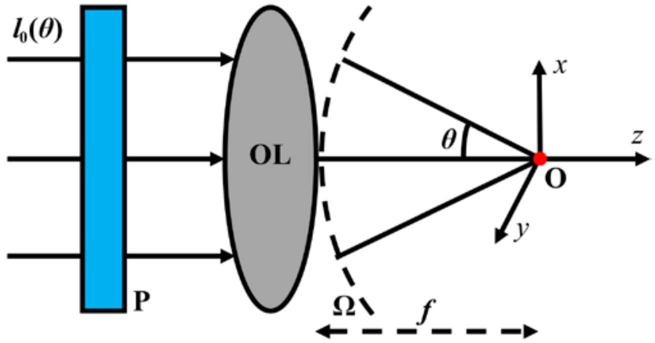

Supplementary Figure 2 Schematic of the focusing system. Ω is the focal sphere, with its center at O and a radius *f*, namely, the focal length of the objective lens (OL). P is the pupil filter in the wavefront of the lens. θ is the convergent angle. $l_0(\theta)$ denotes the electric amplitude of incident Gaussian beams.

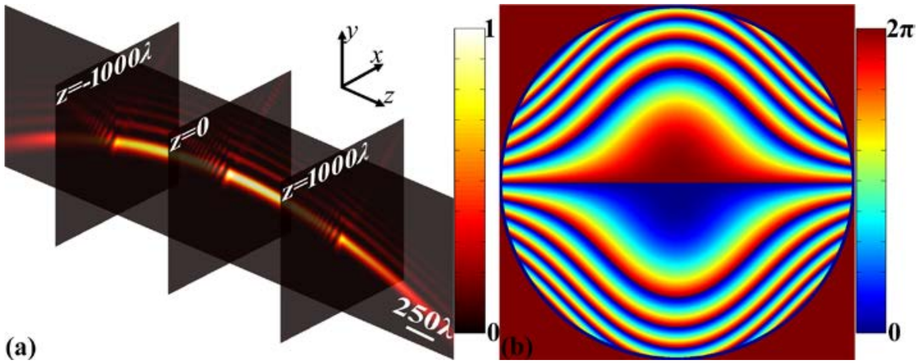

Supplementary Figure 3 Normalized light intensity of quasi-Airy beam. Quasi-Airy beam (a) is generated by the cubic phase (b), the parameters of which are $\eta = 5$, $\sigma = 3$ and $\varphi_0 = 0.75\pi$.

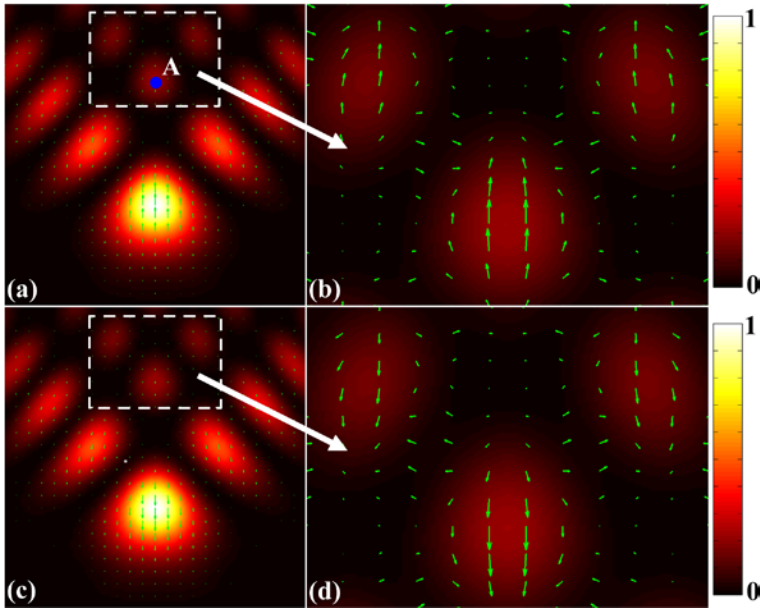

Supplementary Figure 4 Light intensities of quasi-Airy beam in two symmetrical planes. (a, b) are the light intensities in the z=–1000λ, while their counterparts in z=1000λ plane are shown in (c, d). The corresponding transverse energy fluxes are indicated by the green arrows. Point A denotes the first sidelobe of the light beam.

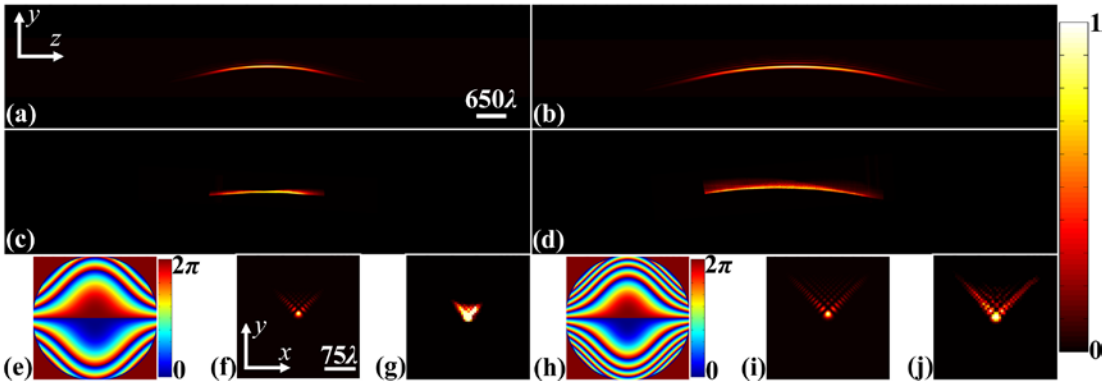

Supplementary Figure 5 Quasi-Airy beams with different η under the condition of NA=0.095 and $\sigma = 3$. (a) Quasi-Airy beam with $\eta = 3$; (b) Quasi-Airy beam with $\eta = 5$. All are generated by the phases (e, h), respectively. (f, i) show the light intensities of both light beams in the z=0 plane. (c, d) and (g, j) present the corresponding experimental results. The FWHMs of (a) and (b) are theoretically approximately 1515.6λ and 2560λ, respectively, while their experimental results are (c) 1522.1λ and (d) 2544.9λ, respectively. The light intensities of quasi-Airy beams are normalized to a unit value, which are indicated by the color bar. The phase scales of (e, h) are 0~2π.

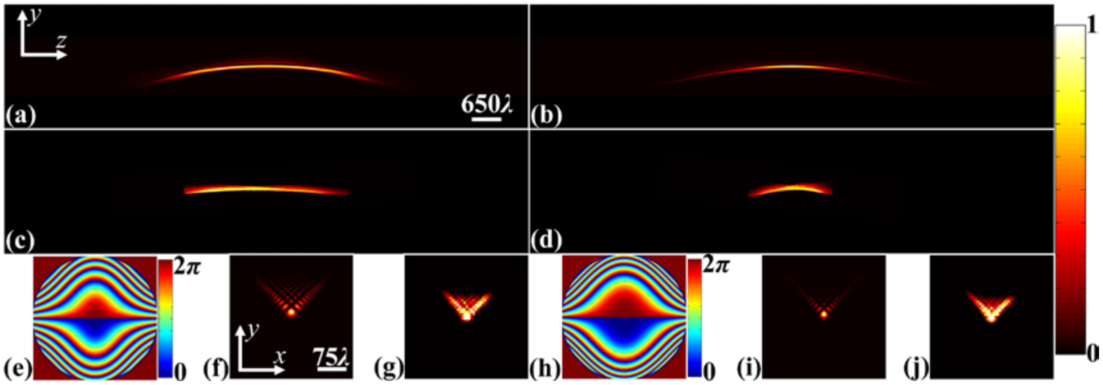

Supplementary Figure 6 Quasi-Airy beams with different σ under the condition of NA=0.095 and η=5. (a) Quasi-Airy beam with σ=2.6; (b) Quasi-Airy beam with σ=3.6. All are generated by the phases (e, h), respectively. (f, i) show the light intensities of both light beams in the z=0 plane. (c, d) and (g, j) present the corresponding experimental results. The FWHMs of (a) and (b) are theoretically approximately 3084λ and 1411λ, respectively, while their experimental results are (c) 3077.6λ and (d) 1419.3λ, respectively. The light intensities of quasi-Airy beams are normalized to a unit value, which are indicated by the color bar. The phase scales of (e, h) are 0~2π.

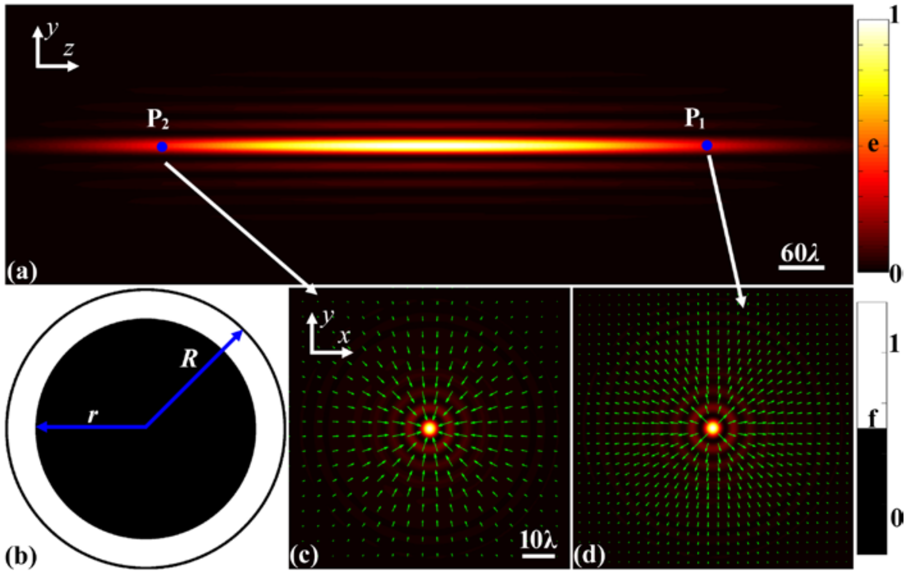

Supplementary Figure 7 Bessel beam generated using high-pass pupil filter. (a) Light intensity of Bessel beam in y-z plane; (b) High-pass pupil filter with a ratio of the inner to outer ring radius r/R=0.8; (c, d) Light intensities and energy fluxes (green arrows) at points P_2, P_1 in the z=–300λ, 300λ planes, respectively. The light intensities of Bessel beam are normalized to a unit value, which are indicated by the color bar (e). The color bar (f) indicates the transmittance of high pass pupil filter (b).

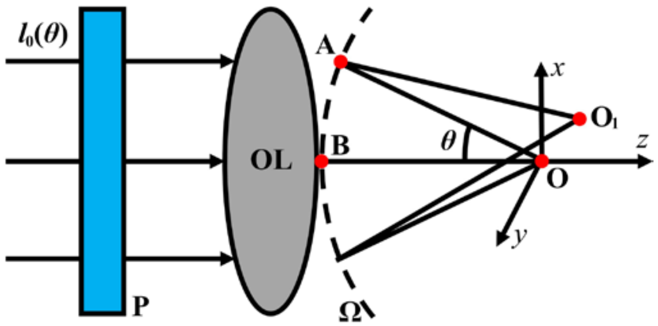

Supplementary Figure 8 Schematic of deriving optical pen in focusing system. Ω is the focal sphere, with its center at O and a radius *f*. A, B are the off- and on-axis points in Ω . O_1 is an arbitrary point in the focal region. P is the pupil filter in the wavefront of the objective lens (OL). $l_0(\theta)$ denotes the electric amplitude of incident Gaussian beams and θ is the convergent angle of OL.

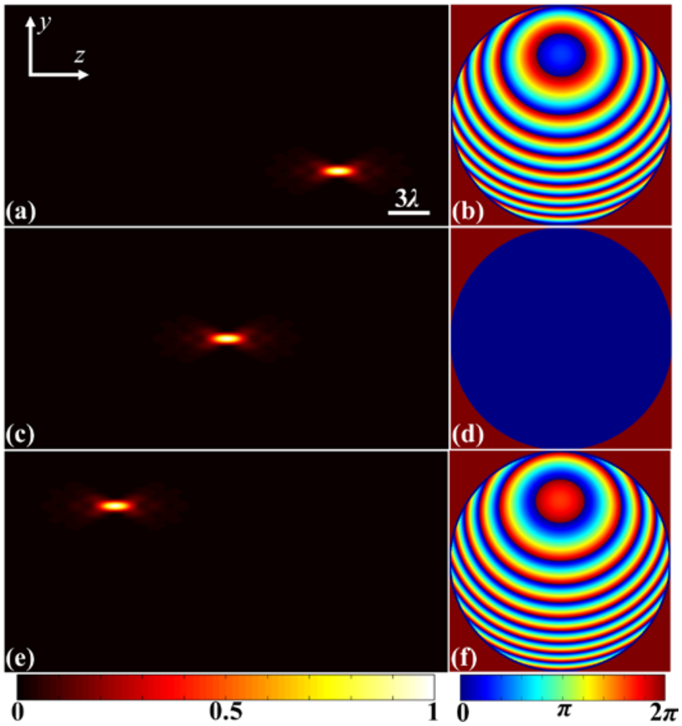

Supplementary Figure 9 Shifting foci in the *y*-*z* plane. The locations of foci in (a, c, e) are adjusted by the phases in (b, d, f), the parameters of which are N=1, s_1=1, δ=0 , x=0, and (a) y=–5, z=10; (c) y=0, z=0; and (e) y=5, z=–10.

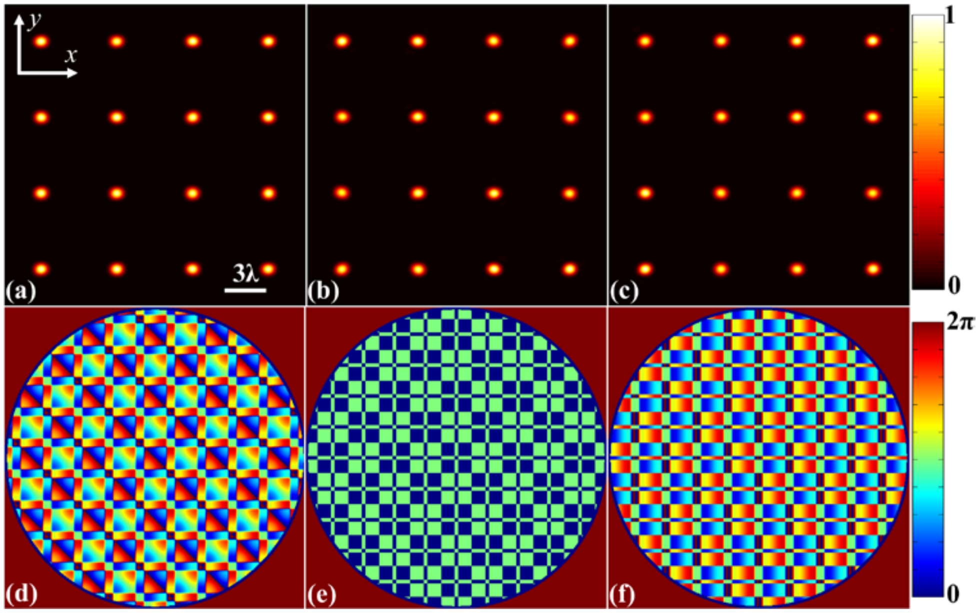

Supplementary Figure 10 Focal arrays in the focal plane. Identical 4×4 focal arrays in (a-c) can be created by the different phases in (d-f), respectively. Here, these focal arrays are realized by multiplying two phases, which yield 1×4 focal arrays along the *x*- and *y*-axis. Thus, N=4, z_j=0, and δ_j=0. For 1×4 foci along the *x*-axis, y_j=0, with the foci located at $x_1 = -3$, $x_2 = 3$, $x_3 = -9$ and $x_4 = 9$. For 1×4 foci along the *y*-axis, x_j=0, with the foci located at $y_1 = -3$, $y_2 = 3$, $y_3 = -9$ and $y_4 = 9$. Three pupil filters for the 1×4 focal array along the *x*- and *y*-axis are obtained, with s_1=1.05, s_2=0.7, s_3=0.92, s_4=–0.28; s_1=s_2=–0.885, s_3=s_4=1; and s_1=–1.05, s_2=0.7, s_3=0.95, s_4=0.33, which are denoted as $l_{x1} \sim l_{x3}$ for the *x*-axis and $l_{y1} \sim l_{y3}$ for the *y*-axis. Finally, the phases for (d-f) are (d) $l_{x1} \times l_{y1}$; (e) $l_{x2} \times l_{y2}$; and (f) $l_{x3} \times l_{y2}$.

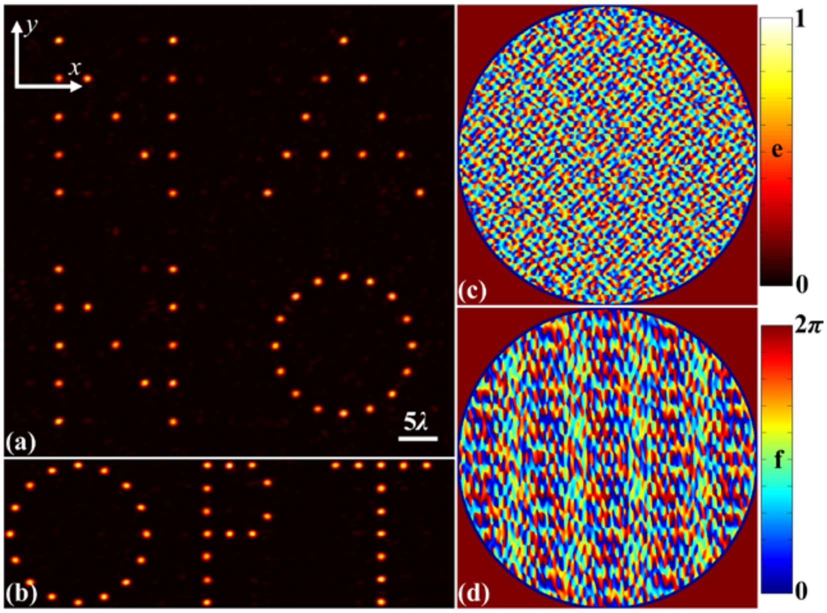

Supplementary Figure 11 Arbitrary focal pattern in the focal plane. NANO (a) and OPT (b) are obtained by the phases (c, d), respectively. The light intensities in (a, b) are normalized to a unit value, which are indicated by the color bar (e). The color bar (f) shows the phase scale of (c, d). The parameters can be found in the Supplementary Note 3.

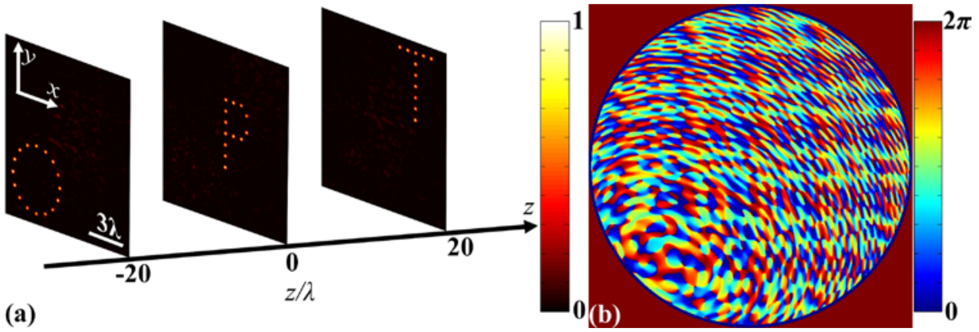

Supplementary Figure 12 Arbitrary focal pattern in the different z planes. (a) O, P, and T are generated in the z=–20λ, z=0 and z=20λ planes simultaneously using the phase (b). The parameters can be found in the Supplementary Note 3.

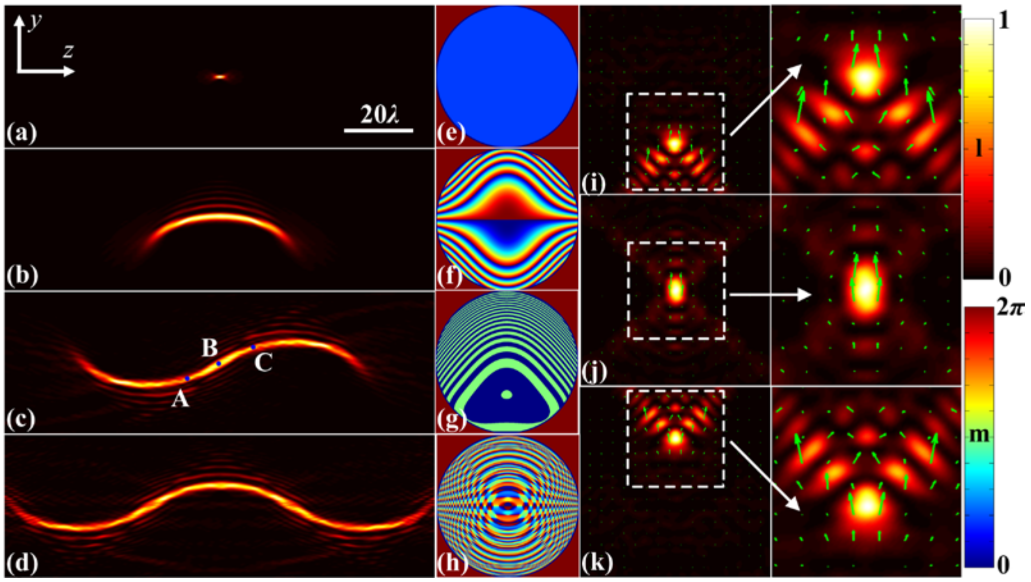

Supplementary Figure 13 UAD light beams with different numbers of energy oscillations under the condition of NA=0.8. (a) zero oscillations (2.34λ); (b) one oscillation (31.96λ); (c) two oscillations (70.56λ); (d) three oscillations (99.50λ). All are generated by the phases (e-h), respectively. The parameters can be found in Supplementary Table 1. (i-k) Light intensities and energy fluxes (green arrows) of points A, B, and C in the z=–10λ, 0, and 10λ planes. Here, all light intensities are normalized to a unit value, which are indicated by the color bar (l). The color bar (m) shows the phase scale of (e-h).

Supplementary Table 1: UAD Light Beams under the condition of NA=0.8

N	y_1	y_2	y_3	z_1	z_2	z_3
1	0			0		
1	0			0		
2	–4.92	4.92		–19.505	19.505	
3	–4.92	4.92	–4.92	–39.12	0	39.12
η_1	η_2	η_3	δ_1	δ_2	δ_3	Parameter
0						NA=0.8
5						φ_0=0.75π
–5	5		–0.95π	0.95π		x_j=0
–5	5	–5	0.26π	0	–0.26π	s_j=1

Supplementary Note 1: Definition of Finite Power for an Anti-Diffracting Light Beam

In this paper, the concept of finite power is adopted as an intrinsic attribute of the anti-diffracting light beams. Note that the finite power is prevalent in the regime of anti-diffracting light beams: see references [1-4]. To be consistent with these references, the power for an anti-diffracting light beam is defined as the integration of the amplitude squared along the propagation trajectory. Therefore, for an ideal anti-diffracting light beam with infinite propagation distance, infinite power is needed to maintain its shape in free space; For a quasi- anti-diffracting light beam, it only possesses finite power in free space, resulting in a finite anti-diffracting distance.

Taking the ideal Bessel beam and Airy beam as example. As shown in Supplementary Figure 1(a), an ideal Bessel beam with an amplitude proportional to $J_n(k_r r)\exp(-ik_z z)$ can preserve its shape infinitely without divergence during propagation in free space. Thus, the power of the ideal Bessel beam can be obtained by [1]

$$I_B = \left| \iint_{-\infty}^{+\infty} J_n(k_r r)\exp(-ik_z z) drdz \right|^2 \to \infty \quad . \tag{1}$$

Similarly, the power of the ideal Airy beam in Supplementary Figure 1(b) can be obtained by [2]

$$I_A = \left| \iint_{-\infty}^{+\infty} Ai(s - \xi^2/4)\exp[i(s\xi/2 - \xi^3/12)] dxdz \right|^2 \to \infty \tag{2}$$

where $s = x/x_0$, $\xi = z/kx_0^2$, and x_0 is an arbitrary constant.

Since the Bessel and Airy function in Supplementary Equations 1, 2 are not square integrable, both light beams possess infinite power in free space. However, such infinite power can only be achieved by the infinite aperture of the lens. In practice, finite apertures can only transfer finite power to an anti-diffracting light beam, thereby leading to a finite anti-diffracting distance in free space. For this reason, the creation of ultralong anti-diffracting (UAD) light beam is always considered to be impossible in free space.

Supplementary Note 2: Energy Oscillation of Anti-Diffracting Light Beams

Anti-diffracting light beams are special solutions of the Helmholtz equation (HE). According to their mathematical forms, these light beams can infinitely preserve their shapes without divergence during propagation in free space [1-4]. However, due to finite power in free space, only light beams with finite anti-diffracting distances can be obtained. It is well known that anti-diffracting light beams are composed of a mainlobe and sidelobes. As the light intensity of sidelobes increases, the anti-diffracting distance increases accordingly. Up to now, little is known about the mechanism underlying this physical phenomenon. To reveal this mechanism, the roles of the sidelobes and mainlobe in counteracting the diffraction effect in free space must be determined.

In theory, anti-diffracting light beams are generally obtained by the Fourier transformation of their Fourier spectra [1-4]. The process of Fourier transformation can be accomplished by an objective lens in Supplementary Figure 2 [5, 6]. For example, a quasi-Airy beam can be obtained by focusing a Gaussian beam modulated using a cubic phase plate [2], whereas a Bessel beam can be created by the Fourier transformation of a Gaussian beam modulated using a high pass filter [1]. Based on the Debye vectorial diffractive theory, the electric and magnetic fields of an anti-diffracting light beam near the focus can be expressed as [7, 8]

$$\mathbf{E}\ \mathbf{or}\ \mathbf{H} = -\frac{iA}{\pi}\int_0^{2\pi}\int_0^{\alpha} \sin\theta \cos^{1/2}\theta T_c l_0(\theta)\mathbf{V}\exp(-ik\mathbf{s}\bullet\boldsymbol{\rho})d\theta d\varphi \tag{3}$$

where θ and φ are the convergent angle and azimuthal angle, respectively; and A is a normalized constant. In addition, $\alpha = \arcsin(\mathrm{NA}/n)$, where NA is the numerical aperture of the objective lens, and n is the refractive index in the focusing space. The wavenumber is $k = 2n\pi/\lambda$, where λ is the wavelength of the incident beam, and $\boldsymbol{\rho} = (r\cos\phi, r\sin\phi, z)$ denotes the position vector of an arbitrary field point in the focal region. In the spherical polar coordinates, the sign of the unit vector $\mathbf{s}$ along a ray in the focusing space is incorrect, as shown in Fig. 1 of Ref.[7]; i.e., $\mathbf{s} = (-\sin\theta\cos\varphi, -\sin\theta\sin\varphi, \cos\theta)$ is used instead of $(\sin\theta\cos\varphi, \sin\theta\sin\varphi, \cos\theta)$. Using $\mathbf{s}$ with the opposite sign will lead to symmetry between the calculated and experimental results about the focal plane. In addition, T_c is the Fourier spectrum of the desired anti-diffracting light beam, and $l_0(\theta)$ is the electric amplitude of the incident Gaussian beam, which can be expressed as

$$l_0(\theta) = \exp[-(\beta_0 \frac{\sin\theta}{\sin\alpha})^2] \tag{4}$$

where β_0 is the ratio of the pupil radius to the incident beam waist.

In Supplementary Equation 3, **V** represents the propagation unit vector of the incident beam right after having passed through the lens. Here, we take a linearly polarized beam as an example to generate an anti-diffracting light beam. Thus, the electric vector $\mathbf{V}_e$ and magnetic vector $\mathbf{V}_m$ can be written as [7]

$$\mathbf{V}_e = \begin{bmatrix} \cos\theta + (1-\cos\theta)\sin^2\varphi \\ -(1-\cos\theta)\sin\varphi\cos\varphi \\ \sin\theta\cos\varphi \end{bmatrix}; \ \mathbf{V}_m = \begin{bmatrix} -(1-\cos\theta)\sin\varphi\cos\varphi \\ 1-(1-\cos\theta)\sin^2\varphi \\ \sin\theta\sin\varphi \end{bmatrix}. \tag{5}$$

Eventually, the light intensity distribution of an anti-diffracting light beam can be obtained using $I = |\mathbf{E}|^2$. Moreover, the time-averaged Poynting vector, namely, the energy flux, can be obtained using [9]

$$\langle \mathbf{S} \rangle = \frac{c}{4\pi} \mathrm{Re}\left(\mathbf{E} \times \mathbf{H}^*\right) \tag{6}$$

where c is the velocity of light in a vacuum, and the asterisk denotes the operation of complex conjugation. According to Supplementary Equation 6, one can flexibly explore the energy process of anti-diffracting light beams during propagation in free space.

Suppose that $\mathrm{P}_1(x, y, z)$ and $\mathrm{P}_2(x, y, -z)$ are two symmetrical points on the anti-diffracting light beam. The electric and magnetic fields of these points can be expressed as [7]

$$\begin{aligned} E_x(\mathrm{x,y,-z}) &= -E_x^*(\mathrm{x,y,z}) \quad & H_x(\mathrm{x,y,-z}) &= -H_x^*(\mathrm{x,y,z}) \\ E_y(\mathrm{x,y,-z}) &= -E_y^*(\mathrm{x,y,z}); & H_y(\mathrm{x,y,-z}) &= -H_y^*(\mathrm{x,y,z}). \\ E_z(\mathrm{x,y,-z}) &= E_z^*(\mathrm{x,y,z}) & H_z(\mathrm{x,y,-z}) &= H_z^*(\mathrm{x,y,z}) \end{aligned} \tag{7}$$

By substituting Supplementary Equation 7 into 6, the relationship of transverse energy fluxes between $\mathrm{P}_1(x, y, z)$ and $\mathrm{P}_2(x, y, -z)$ can be expressed as

$$\langle \mathbf{S} \rangle_{tP_1} = -\langle \mathbf{S} \rangle_{tP_2}, \tag{8}$$

which implies that the light beam experiences two inverse energy processes that transfer the energy from $\langle \mathbf{S} \rangle_{tP_2}$ to $\langle \mathbf{S} \rangle_{tP_1}$ during propagation in free space. Without a loss of generality, $\langle \mathbf{S} \rangle_{tP_2}$ and $\langle \mathbf{S} \rangle_{tP_1}$ are the energy charge and energy discharge, respectively.

Energy charge and discharge compose an entire energy oscillation, which is a directional energy flux that confines the energy of anti-diffracting light beams into an interaction between the mainlobe and

sidelobes when propagating in free space. Thus, the light beam would not diverge freely as observed with a Gaussian beam in free space. If only energy oscillation occurs, an anti-diffracting light beam can preserve its shape without divergence in free space. Even when encountering an obstacle, the mainlobe can carry out self-healing with the power from the sidelobes [10]. That is why an anti-diffracting light beam is naturally composed of a mainlobe and sidelobes. Moreover, since all anti-diffracting light beams can be created by focusing their corresponding Fourier spectra in Supplementary Figure 2, energy oscillation is therefore a general property shared by all anti-diffracting light beams.

Energy oscillation mechanism of an Airy beam

Although energy oscillation is a general property shared by all anti-diffracting light beams, different light beams exhibit different forms of energy oscillation, which are mainly determined by the Fourier spectrum T_c. For example, the cubic phase is the Fourier spectrum of Airy beam, the transmittance of which can be written as [2, 3]

$$T_c = \exp\left\{ i\eta \frac{k\sin^\sigma \theta}{\sin^\sigma \alpha} \left[\sin^3(\varphi+\varphi_0) + \cos^3(\varphi+\varphi_0) \right] \right\} \tag{9}$$

where η and σ are the parameters that control the period and phase distribution of the cubic phase plate, respectively. Typically, if $\sigma = 3$, then T_c denotes a standard cubic phase, and the entire phase can be rotated by an angle φ_0. Accordingly, a quasi-Airy beam generated using the cubic phase of $-\eta$ can simply be obtained by rotating the cubic phase of η with $\varphi_0=\pi$. That is, light beams with $\pm\eta$ are symmetrical about the optical axis, thereby leading to

$$\langle \mathbf{S}_\eta \rangle_{tP_1} = -\langle \mathbf{S}_{-\eta} \rangle_{tP_1} \tag{10}$$

where $\langle \mathbf{S}_\eta \rangle_{tP_1}$ and $\langle \mathbf{S}_{-\eta} \rangle_{tP_1}$ are the transverse energy fluxes of point $\mathrm{P}_1(x, y, z)$ for η and $-\eta$, respectively.

From Supplementary Equations 8 and 10, the transverse energy flux relationship between quasi-Airy beams with $\pm\eta$ can be simplified to

$$\langle \mathbf{S}_\eta \rangle_{tP_1} = \langle \mathbf{S}_{-\eta} \rangle_{tP_2} = -\langle \mathbf{S}_\eta \rangle_{tP_2} = -\langle \mathbf{S}_{-\eta} \rangle_{tP_1} \quad , \tag{11}$$

where the energy charge $\left\langle \mathbf{S}_{\eta} \right\rangle_{tP_2}$ is equal to the energy discharge $\left\langle \mathbf{S}_{-\eta} \right\rangle_{tP_1}$, and the energy discharge $\left\langle \mathbf{S}_{\eta} \right\rangle_{tP_1}$ is equal to the energy charge $\left\langle \mathbf{S}_{-\eta} \right\rangle_{tP_2}$. Thus, quasi-Airy beams with $\pm\eta$ can be considered a pair of mutually complementary modes in free space.

Energy interaction between the mainlobe and sidelobes

In the following simulations and experiments, NA=0.095, n=1, and $\beta_0 = 1$. The unit of length in all figures is the wavelength λ, and the light intensity is normalized to the unit value. The anti-diffracting distance of the UAD light beam is evaluated with the FWHM (full width at half maximum).

The quasi-Airy beam in Supplementary Figure 3(a) generated by the cubic phase with the parameters $\eta = 5$, $\sigma = 3$ and φ_0=0.75π in Supplementary Figure 3(b) represents one entire energy oscillation in free space, which is composed of energy charge when z<0 and discharge when z>0. Energy charge and discharge imply two different energy processes. In the process of energy charge when z<0, the energy is transported from the mainlobe to the sidelobes as shown in Supplementary Figure 4(a, b). Thus, the mainlobe is the energy source of the sidelobes, and the energy of the mainlobe tends to be stored in the sidelobes instead of diverging as observed with a Gaussian beam. Compared with energy charge, the light beam when z>0 experiences a totally different energy process, namely, energy discharge. Due to the inverse energy flux in Supplementary Figure 4(c, d), the energy is transported from the sidelobes to the mainlobe. In this case, the sidelobes are the energy sources of the mainlobe while the mainlobe becomes an energy consumer. During propagation when z>0, the mainlobe endures an energy loss caused by the diffraction effect. However, this energy can be replenished by the power of the sidelobes; thus, the light beam can remain anti-diffracting. Even when encountering an obstacle, the light beam can carry out self-healing through the power of the sidelobes [10].

Due to the energy oscillation mechanism, the power of the quasi-Airy beam is confined to the interplay between the mainlobe and sidelobes. Therefore, the light beam can propagate without significant divergence in free space. However, this confinement cannot be limitless. Finite power in free space can only support a finite energy charge when z<0, thus leading to only a finite energy discharge when z>0. When the power stored in the sidelobes is exhausted, the light beam can no longer maintain its shape at z>0, and the diffraction effect eventually dominates. Consequently, the light beam can propagate only a finite anti-diffracting distance in free space. In addition, the power of energy charge is equal to that of energy discharge because of energy conversation, thereby generating an equivalent non-diffractive distance when z<0 and z>0. For this reason, a quasi-Airy beam possesses a symmetrical

trajectory as shown in Supplementary Figure 3(a).

Throughout the entire energy oscillation process, the z=0 plane is the inflection plane in which the role of the mainlobe changes from the energy source to the energy consumer. Specifically, when z<0, the sidelobes can receive power from the mainlobe continually during the energy charge process. However, once the sidelobes attain the maximum energy capacity, energy charge can no longer be conducted, and the sidelobes begin providing power to the mainlobe when z>0. Thus, the sidelobes have maximum light intensity in the z=0 plane. Suppose that I_s is the light intensity of sidelobe A in the z=0 plane, as shown in Supplementary Figure 4(a). In principle, a larger I_s indicates stronger energy oscillation, thereby leading to a longer anti-diffracting distance of the light beam in free space. That is, if I_s can be adjusted, then the strength of the energy oscillation along with the non-diffractive distance can be controlled accordingly.

Adjusting the Strength of Energy Oscillation

Two methods are proposed to control the strength of energy oscillation via I_s: adjust the period of cubic phase η while $\sigma = 3$, and manipulate the cubic phase distribution via σ while keeping the period η unchanged. For the first method, quasi-Airy beams with different values of η are generated as shown in Supplementary Figure 5, where $\eta = 3$ in (a, f) and $\eta = 5$ in (b, i). Supplementary Figures 5(e, h) depict their corresponding cubic phase plates. The theoretical results show that I_s increases with the period of the cubic phase η, where I_s=0.213 for $\eta = 3$ [Supplementary Figure 5(f)] and I_s=0.239 for $\eta = 5$ [Supplementary Figure 5(i)]. Therefore, a longer non-diffractive distance can be achieved when η is larger. The FWHMs of Supplementary Figure 5(a) and (b) are 1515.6λ and 2560λ, respectively. By adopting the same experimental setup as in Fig. 1 of the main text, quasi-Airy beams with $\eta = 3$ [Supplementary Figure 5(c)] and $\eta = 5$ [Supplementary Figure 5(d)] are created under the condition of NA=0.095. The FWHMs are 1522.1λ and 2544.9λ, which are coincident with those of Supplementary Figure 5(a, b) in theory.

As for the second one, Supplementary Figure 6 shows the light intensity of quasi-Airy beams with $\sigma = 2.6$ (a, f) and $\sigma = 3.6$ (b, i) when $\eta = 5$. As shown in Supplementary Figure 6(f, i), I_s increases as σ decreases, with I_s=0.355 [Supplementary Figure 6(f)] and I_s=0.239 [Supplementary Figure 6(i)]. Similarly, the non-diffractive distance of $\sigma = 2.6$ is much longer than that of $\sigma = 3.6$. In the experiment, the quasi-Airy beams shown in Supplementary Figure 6(a, f) and (b, i) are created by coding the phase of Supplementary Figure 6(e, h) in the SLM shown in Fig. 1 of the main text. The FWHM values for

both beams are 3077.6λ [Supplementary Figure 6(c)] and 1419.3λ [Supplementary Figure 6(d)], respectively, which fit well with those of $\sigma = 2.6$ (3084λ) and 3.6 (1411λ) in theory. Although both methods mentioned above are capable of adjusting the energy strength via the light intensity of sidelobe I_s, neither method can strengthen the energy oscillation without limitation, which makes the ideal Airy beam impossible in practice. However, this new mechanism still offers the possibility of breaking through the light beam limitation of finite non-diffractive distance in free space.

Energy oscillation mechanism of a Bessel beam

Supplementary Figure 7 presents the light intensity and energy flux of a Bessel beam, which is generated by focusing a linearly polarized Gaussian beam with the modulation of a high-pass pupil filter in Supplementary Figure 7(b) [1]. Here, the transmission of this pupil filter can be expressed as

$$T_h = \begin{cases} 1 & r/R \geq 0.8 \\ 0 & 0 \leq r/R < 0.8 \end{cases}, \tag{12}$$

where r and R are the radii of the inner and outer ring of the high-pass pupil filter, respectively. By substituting T_c for T_h in Supplementary Equation 3, the light intensity and energy flux of the Bessel beam can be calculated using Debye vectorial diffractive theory. In the following simulations, NA=0.095, n=1, and $\beta_0 = 1$. The unit of length in all figures is the wavelength λ, and the light intensity is normalized to the unit value.

As shown in Supplementary Figure 7(c, d), a Bessel beam can be divided into two parts according to the energy flux: energy charge when z<0 and energy discharge when z>0. For the light beam when z<0, the energy is transported from the sidelobes to the central mainlobe radially. Thus, the sidelobes are the energy source of the mainlobe, as shown in Supplementary Figure 7(c). Even when encountering an obstacle, the light beam can carry out self-healing [11]. In contrast to the light beam when z<0, the light beam when z>0 experiences an inverse energy process. As shown in Supplementary Figure 7(d), the energy is transported from the mainlobe to the sidelobes radially. In this case, the mainlobe turns into an energy source, while the sidelobes serve as the energy consumer of the mainlobe. Via this energy interaction between the mainlobe and sidelobes, the Bessel beam can preserve its shape without a significant divergence in free space. Similar to the quasi-Airy beam in Supplementary Figure 4, finite power in free space can support only a finite energy oscillation, thus leading to a finite anti-diffracting distance of the Bessel beam.

Supplementary Note 3: Derivation of the Optical Pen

Supplementary Figure 8 presents a schematic of deriving optical pen in focusing system. A collimate incident vector beam propagating along the +*z*-axis goes through the pupil filter P before being focused by a lens obeying the sine condition. Ω is the focal sphere, and its center is at O and its radius is *f*, which is the focal length of the lens. O_1 is an arbitrary point in the focal region of the lens. In principle, only one focus is located at the focal point O, where constructive interference can occur only because of the equivalent optical paths of the light beams between the points in Ω and point O. If constructive interference does not occur at point O but rather at arbitrary point O_1, the focus is generated at point O_1 instead of at O. To this end, the optical path difference (OPD) for the light beams between the points in Ω and O_1 must be compensated by the phase of pupil filter P.

As shown in Supplementary Figure 8, the OPD for the light beams between the points in Ω and O_1 can be simplified to L_2-L_1, where L_1 and L_2 are the light paths AO_1 and BO_1, respectively. Here, in the cylindrical coordinate system, the points A, B, O and O_1 can be expressed as $(f\sin\theta\cos\varphi, f\sin\theta\sin\varphi, -f\cos\theta)$, $(0,0,-f)$, $(0,0,0)$ and $(\rho\cos\varphi_s, \rho\sin\varphi_s, z)$, respectively. The light paths L_1 and L_2 can be described as

$$\begin{aligned} L_1 &= \sqrt{\left(f\sin\theta\cos\varphi-\rho\cos\varphi_s\right)^2+\left(f\sin\theta\sin\varphi-\rho\sin\varphi_s\right)^2+(f\cos\theta+z)^2} \\ &= \sqrt{f^2+\rho^2+z^2-2f\rho\sin\theta\cos(\varphi-\varphi_s)+2fz\cos\theta} \end{aligned} \tag{13}$$

$$\begin{aligned} L_2 &= \sqrt{\left(\rho\cos\varphi_s\right)^2+\left(\rho\sin\varphi_s\right)^2+(-f-z)^2} \\ &= \sqrt{\rho^2+f^2+z^2+2fz} \end{aligned} \quad . \tag{14}$$

The OPD for the light beams between the points in Ω and O_1 can be calculated as

$$\Delta s = L_2 - L_1 = \frac{2\rho\sin\theta\cos(\varphi-\varphi_s)+2z(1-\cos\theta)}{\sqrt{\eta_\rho^2+1+\eta_z^2+2\eta_z}+\sqrt{1+\eta_\rho^2+\eta_z^2-2\eta_\rho\sin\theta\cos(\varphi-\varphi_s)+2\eta_z\cos\theta}} \tag{15}$$

where θ and φ are the convergent angle and azimuthal angle, respectively; $\eta_z = z/f$; and $\eta_\rho = \rho/f$. Since O_1 is an arbitrary point in the vicinity of the focus, $\rho, z << f$. That is, $\eta_\rho, \eta_z \approx 0$. Finally, Supplementary Equation 15 can be simplified to

$$\Delta s = \rho\sin\theta\cos(\varphi-\varphi_s)+z(1-\cos\theta) \quad . \tag{16}$$

To generate a focus at O_1, Δs must be compensated by the pupil filter P. Thus, $\Delta s_p = -\Delta s$, where

Δs_p is the OPD induced by the pupil filter P. According to the relationship between the phase and OPD, the phase of pupil filter P is $\psi = k\Delta s_p$, where $k = 2\pi n/\lambda$ is the wavenumber, and n is the refractive index in the focusing space. Consequently, the transmittance of pupil filter P for one single focus can be expressed as $T = \exp(i\psi)$. If multiple focuses occur in the focal region, the transmittance of pupil filter P can be further transformed into

$$T = \sum_{j=1}^{N} s_j \exp(i\psi_j) \tag{17}$$

where N is the number of foci and j denotes the j-th focus. As shown in Supplementary Equation 17, the pupil filter P requires both amplitude and phase modulation for the incident light beam. In practice, amplitude modulation always leads to low light transformation efficiency and is difficult to implement. However, this problem can easily be solved by extracting only the phase of pupil filter P in Supplementary Equation 17. Finally, the phase-only pupil filter P can be obtained using

$$T = \exp\left\{i\left[\text{Phase}\left(\sum_{j=1}^{N} s_j \exp[i(\psi_j + \delta_j)]\right)\right]\right\} \tag{18}$$

Here, we refer to this pupil filter as the optical pen. For the sake of simplicity, this optical pen can also be expressed as

$$T = \exp\left\{i\left[\text{Phase}\left(\sum_{j=1}^{N} \text{PF}(s_j, x_j, y_j, z_j, \delta_j)\right)\right]\right\} \tag{19}$$

where x_j, y_j, z_j are the positions of the j-th focus in the focal region corresponding to ρ, φ_s, z in Supplementary Equation 16; and s_j and δ_j are the two weight factors that are responsible for adjusting the amplitude and phase of the j-th focus, respectively.

Manipulation of light field via the optical pen

Note that the pupil filter P developed by the OPD compensation is valid for an arbitrary incident vector beam. Here, we take only a linearly polarized beam as an example to verify the function of the optical pen. Based on Debye vectorial diffractive theory, the light intensity in the vicinity of the focus can be obtained using Supplementary Equation 3. In the following simulations, NA=0.8, n=1, and $\beta_0 = 1$. The unit of length in all figures is the wavelength λ, and the light intensity is normalized to the unit value.

In the case of $N = 1$, only one focus is obtained in the focal region, and its position can be adjusted in three dimensions using the optical pen. As shown in Supplementary Figure 9, foci with different positions in the y-z plane are realized with the different phases of pupil filter P [namely, Supplementary

Figure 9(b, d, f)], the parameters of which are $N=1$, $s_1=1$, $x_1=1$, $\delta=0$; (a, b) $y_1=-5, z_1=10$, (c, d) $y_1=0, z_1=0$, and (e, f) $y_1=5, z_1=-10$. Compared with the original focus in Supplementary Figure 9(c), the shape of the focus remains invariant while the position can be adjusted freely in the focal region.

For an optical pen, the focal region can be considered a drawing board on which an arbitrary pattern can be realized by precisely controlling the number and position of foci and their corresponding weight factors in Supplementary Equation 19. The size of the optical pen is determined by the focus of the incident linearly polarized beam, which is relevant to the NA. For one particular light pattern, the number and position of foci determine the shape of the light pattern, which can be manipulated using the parameters N, x_j, y_j, z_j. Once the shape is confirmed, the amplitude and phase of each focus must be adjusted by s_j and δ_j so that the desired light pattern can be realized in the focal region.

For the same light pattern, which is determined only by the shape, the number and position of foci are identical. However, the weight factors s_j and δ_j have countless possibilities. As shown in Supplementary Figure 10, a 4×4 focal array in the *x*-*y* plane can be realized with the different phases shown in Supplementary Figure 10(d-f), respectively. Supplementary Figure 10(d-f) can be obtained by multiplying two phases, which yields a 1×4 focal array along the *x*- and *y*-axis. Thus, $N=4$, $z_j=0$ and $\delta_j=0$. For the 1×4 foci along the *x*-axis, $y_j=0$ and the foci are located at $x_1=-3$, $x_2=3$, $x_3=-9$ and $x_4=9$. For the 1×4 foci along the *y*-axis, $x_j=0$ and the foci are located at $y_1=-3$, $y_2=3$, $y_3=-9$ and $y_4=9$. The distance between each focus along the *x*- and *y*-axis is 6λ. The only difference between the phases for the same 1×4 foci is the weight factor s_j. For example, three pupil filters for the 1×4 focal array along the *x*- and *y*-axis are obtained, with $s_1=1.05, s_2=0.7, s_3=0.92, s_4=-0.28$; $s_1=s_2=-0.885, s_3=s_4=1$; and $s_1=-1.05, s_2=0.7,$ $s_3=0.95, s_4=0.33$, which are denoted as $l_{x1} \sim l_{x3}$ for the *x*-axis and $l_{y1} \sim l_{y3}$ for the *y*-axis. Finally, the phases can be obtained as follows: (d) $l_{x1} \times l_{y1}$; (e) $l_{x2} \times l_{y2}$; and (f) $l_{x3} \times l_{y2}$. Clearly, all phases in Supplementary Figure 10(d-f) are different from each other, although they all generate an identical 4×4 focal array in the focal plane. The most typical array is the phase in Supplementary Figure 10(e), which is a common Dammann grating, and it was also obtained in Ref. [12]. In other words, the optical pen in Supplementary Equation 19 represents all possible phases, with only the weight factors being different,

and the Dammann grating is only one of the special solutions. Thus, additional phases can be obtained by adjusting the weight factor s_j.

When generating a light pattern in the focal region, the shape is determined by the number and position of foci, whereas the amplitude and phase of each focus ensure the quality of the entire pattern. By controlling the number and position along with the weight factors s_j and δ_j, more complex focal patterns can be realized. As shown in Supplementary Figure 11(a, b), NANO and OPT are created in the focal plane with the phases in Supplementary Figure 11(c, d). In addition, a three-dimensional focal pattern can also be realized using the optical pen. As shown in Supplementary Figure 12(a), OPT is obtained in the $z=-20\lambda, 0, 20\lambda$ planes simultaneously, with the phase shown in Supplementary Figure 12(b). All parameters for the above phases can be found in the Parameters of Supplementary Figures 11 and 12. Once again, the phases for the generation of focal patterns in Supplementary Figures 11 and 12 are not unique, but all can be obtained using s_j and δ_j in Supplementary Equation 19.

Significance of the Optical Pen

The generation of an ultralong anti-diffracting light beam in free space leads to the precise manipulation of the number, position, amplitude and phase of foci in the focal region. However, such precision cannot be achieved using previous techniques [12-17]. The optical pen is therefore developed to solve this problem. As a versatile optical tool, the optical pen possesses an explicit form (Supplementary Equation 19) that can be used to unify the relationship between the focal pattern and the phase in the entrance plane. By adjusting the parameters of the optical pen, the number, position, amplitude and phase of foci can be adjusted at will in the focal region so that an arbitrary focal pattern can be realized in free space. This advantage makes the optical pen a perfect optical tool for the creation of ultralong anti-diffracting light beams in free space.

Parameters of Supplementary Figures 11 and 12

The parameters of the phase in Supplementary Figure 11(c)

Parameters for O

$$l_O = \sum_{j=1}^{N=16} \mathrm{PF}(s_j, x_j, y_j, 0) \ ,$$

where $x_j = d\cos\phi_j$, $y_j = d\sin\phi_j$ and $d=-9$; $\phi_j = 2\pi(j-1)/N$, $j=1,2,3...N$; $s_{1-4} = s_{6,7} = s_{9-12} = s_{16} = 1$; $s_5 = 0.97$; $s_{8,13} = 1.05$; $s_{14} = 1.15$; $s_{15} = 0.98$.

Parameters for N

$d_2 = -7.5$; $d_3 = -10$;

$$\begin{aligned} l_{N1} = {} & \mathrm{PF}(1, d_2, -d_3, 0) + \mathrm{PF}(0.97, -d_2, -d_3, 0) + \mathrm{PF}(-1, d_2, -d_3/2, 0) + \mathrm{PF}(0.95, d_2/2, -d_3/2, 0) + \\ & \mathrm{PF}(-0.97, -d_2, -d_3/2, 0) + \mathrm{PF}(-0.8, d_2, 0, 0) + \mathrm{PF}(1.05, 0, 0, 0) + \mathrm{PF}(-0.8, -d_2, 0, 0) + \\ & \mathrm{PF}(-0.85, d_2, d_3/2, 0) + \mathrm{PF}(0.95, -d_2/2, d_3/2, 0) + \mathrm{PF}(-0.88, -d_2, d_3/2, 0) + \mathrm{PF}(0.88, d_2, d_3, 0) + \\ & \mathrm{PF}(1, -d_2, d_3, 0) + \mathrm{PF}(-0.1, 0, 15, 0) + \mathrm{PF}(-0.1, -d_2/2, -d_3, 0) \end{aligned} ;$$

$$\begin{aligned} l_{N2} = {} & \mathrm{PF}(0.98, d_2, -d_3, 0) + \mathrm{PF}(0.93, -d_2, -d_3, 0) + \mathrm{PF}(-0.88, d_2, -d_3/2, 0) + \mathrm{PF}(0.95, d_2/2, -d_3/2, 0) + \\ & \mathrm{PF}(-0.85, -d_2, -d_3/2, 0) + \mathrm{PF}(-0.8, d_2, 0, 0) + \mathrm{PF}(1.05, 0, 0, 0) + \mathrm{PF}(-0.8, -d_2, 0, 0) + \\ & \mathrm{PF}(-0.91, d_2, d_3/2, 0) + \mathrm{PF}(0.943, -d_2/2, d_3/2, 0) + \mathrm{PF}(-0.93, -d_2, d_3/2, 0) + \mathrm{PF}(0.9, d_2, d_3, 0) + \\ & \mathrm{PF}(1, -d_2, d_3, 0) + \mathrm{PF}(-0.2, -d_2/2, d_3, 0) \end{aligned} ;$$

Parameters for A

$d_4 = -10$; $d_5 = -7.5$;

$$\begin{aligned} l_A = {} & \mathrm{PF}(0.83, 0, -d_4, 0) + \mathrm{PF}(-0.9, d_4/4, -d_4/2, 0) + \mathrm{PF}(-0.9, -d_4/4, -d_4/2, 0) + \mathrm{PF}(0.9, d_4/2, 0, 0) + \\ & \mathrm{PF}(0.9, -d_4/2, 0, 0) + \mathrm{PF}(0.8, d_5, d_4/2, 0) + \mathrm{PF}(0.89, -d_5, d_4/2, 0) + \mathrm{PF}(-0.82, d_4/4, d_4/2, 0) + \\ & \mathrm{PF}(-0.83, -d_4/4, d_4/2, 0) + \mathrm{PF}(0.8, d_4, d_4, 0) + \mathrm{PF}(0.83, -d_4, d_4, 0). \end{aligned} ;$$

Final phase

$d_6 = 15$

$\psi_{pf} = \mathrm{Phase}[\mathrm{PF}(1.3, -d_6, -d_6, 0) l_{N1} + \mathrm{PF}(1.3, -d_6, d_6, 0) l_{N2} + \mathrm{PF}(1, d_6, d_6, 0) l_A + \mathrm{PF}(0.9, d_6, -d_6, 0) l_O]$;

$l_{pf} = \exp(i\psi_{pf})$.

The parameters of the phase in Supplementary Figure 11(d)

Parameters for O

$$l_O = \sum_{j=1}^{N=16} \mathrm{PF}(s_j, x_j, y_j, 0) ,$$

where $x_j = d\cos\phi_j$, $y_j = d\sin\phi_j$; $d = -9$; $\phi_j = 2\pi(j-1)/N, j = 1,2,3...N$; $s_{1,3} = s_{6,7} = 1$; $s_{2,11} = 1.03$;

$s_4 = 1.01$; $s_5 = 1.15$; $s_8 = 1.1$; $s_9 = 1.08$; $s_{10} = 1.05$; $s_{13} = 1.24$; $s_{12,14-16} = 1.02$.

Parameters for P

$$\begin{aligned} l_P = {} & \sum_{j=1}^{7} \mathrm{PF}(s_j, 0, d_j, 0) + \mathrm{PF}(s_8, 3, 0, 0) + \mathrm{PF}(s_9, 6, 0, 0) + \mathrm{PF}(s_{10}, 3, 9, 0) + \mathrm{PF}(s_{11}, 6, 9, 0) + \\ & \mathrm{PF}(s_{12}, 8, 6.75, 0) + \mathrm{PF}(s_{13}, 8, 2.25, 0) + \mathrm{PF}(s_{14}, 3, -9, 0) \end{aligned} ,$$

where $d_j = (j-4)\times 3$; $s_1 = 1.125$; $s_2 = 1.12$; $s_{3,5} = -1.205$; $s_4 = 1.505$; $s_6 = 0.935$; $s_{7,13} = 1.1$; $s_8 = 1.6$;

$s_9 = 1.4$; $s_{10} = 1.45$; $s_{11,12} = 1.2$; $s_{14} = 0.3$.

Parameters for T

$$l_T = \sum_{j=1}^{7} \mathrm{PF}(s_j, 0, d_j, 0) + \sum_{j=8}^{9} \mathrm{PF}(s_j, D_j, 9, 0) + \sum_{j=10}^{11} \mathrm{PF}(s_j, D_{j+1}, 9, 0),$$

where $d_j = (j-4)\times 3$; $D_j = (j-10)\times 3$ and $s_1 = 1.4$; $s_2 = -1.26$; $s_3 = 1.3$; $s_4 = 1.1$; $s_5 = 1.25$; $s_6 = -1.35$; $s_7 = 1.55$; $s_8 = -0.95$; $s_{9,10} = 1.46$; $s_{11} = -1.15$.

Final phase

$$\psi_{pf} = \mathrm{Phase}[\mathrm{PF}(1.45, -20, 0, 0) l_O + \mathrm{PF}(1.1, -3, 0, 0) l_P + \mathrm{PF}(1.1, 20, 0, 0) l_T];$$

$$l_{pf} = \exp(i\psi_{pf}).$$

The parameters of the phase in Supplementary Figure 12

Parameters for O

$$l_O = \sum_{j=1}^{N=16} PF(s_j, x_j, y_j, 0),$$

where $x_j = d\cos\phi_j$, $y_j = d\sin\phi_j$ and $d = -9$; $\phi_j = 2\pi(j-1)/N, j = 1,2,3...N$; $s_1 = 0.95$; $s_{13} = 1.2$; $s_{2,4,6-8,14-16} = 1$; $s_{3,5,9-11} = 1.1$.

Parameters for P

$$l_P = \sum_{j=1}^{7} \mathrm{PF}(s_j, 0, d_j, 0) + \mathrm{PF}(s_8, 3, 0, 0) + \mathrm{PF}(s_9, 6, 0, 0) + \mathrm{PF}(s_{10}, 3, 9, 0) + \mathrm{PF}(s_{11}, 6, 9, 0) + \mathrm{PF}(s_{12}, 8, 6.75, 0) + \mathrm{PF}(s_{13}, 8, 2.25, 0),$$

where $d_j = (j-4)\times 3$; $s_{1,7} = 0.8$; $s_{2,10,11} = 1.3$; $s_3 = -1.3$; $s_4 = 1.6$; $s_5 = -1.4$; $s_{6,9} = 1.2$; $s_8 = 1.4$; $s_{12} = 1.15$; $s_{13} = 1$.

Parameters for T

$$l_T = \sum_{j=1}^{7} \mathrm{PF}(s_j, 0, d_j, 0) + \sum_{j=8}^{9} \mathrm{PF}(s_j, D_j, 9, 0) + \sum_{j=10}^{11} \mathrm{PF}(s_j, D_{j+1}, 9, 0),$$

where $d_j = (j-4)\times 3$, $D_j = (j-10)\times 3$; $s_1 = 1.45$; $s_2 = -1.35$; $s_3 = 1.35$; $s_4 = 0.8$; $s_5 = 1.23$; $s_6 = -1.25$; $s_7 = 1.4$; $s_8 = -1$; $s_{9,10} = 1.3$; $s_{11} = -0.95$.

Final phase

$$\psi_{pf} = \mathrm{Phase}[\mathrm{PF}(1.25, -12, -12, -20) l_O + \mathrm{PF}(0.98, 0, 0, 0) l_P + \mathrm{PF}(1, 12, 12, 20) l_T];$$

$$l_{pf} = \exp(i\psi_{pf}).$$

Supplementary Note 4: UAD light beams under the condition of NA=0.8

Supplementary Figure 13 shows UAD light beams with 0, 1, 2, and 3 energy oscillations in the y-z plane, and they are generated by focusing a linearly polarized Gaussian beam with NA=0.8. The corresponding phases are shown in Supplementary Figure 13(e-h), and the parameters can be found in Supplementary Table 1. Notably, the anti-diffracting distances of the UAD light beams for zero and one energy oscillation are only 2.34λ and 31.96λ, respectively. However, using the multiple energy oscillation mechanism, UAD light beams can be easily achieved by simply increasing the number of energy oscillations via modulation of the optical pen. As shown in Supplementary Figure 13(c, d), the anti-diffracting distances of two and three energy oscillations are 70.56λ and 99.50λ, respectively.

The multiple energy oscillations mechanism can lead to a peculiar energy flux at the switch point between adjacent energy oscillations as indicated by the cross-shaped sidelobes in Supplementary Figure 13(j). The bottom sidelobe corresponds to an energy discharge process similar to that at point A in the initial energy oscillation as shown in Supplementary Figure 13(i). Although the energy discharge at point B will soon be completed, the upper sidelobes can provide an additional energy recharge so that the light beam can propagate further. That is, at point B, the light beam experiences not only an energy discharge in the initial energy oscillation but also an energy charge similar to that at point C in Supplementary Figure 13(k) in the second energy oscillation. Thus, the UAD light beam can maintain its shape without significant divergence over a super-long range.

Supplementary References

1. Durnin, J., Miceli, J. J., Eberly, J. H. Diffraction-free beams. *Phys. Rev. Lett.* **58**, 1499-1501 (1987).
2. Siviloglou, G. A., Broky, J., Dogariu, A., Christodoulides, D. N. Observation of Accelerating Airy Beams. *Phys. Rev. Lett.* **99**, 213901 (2007).
3. Siviloglou, G. A., Christodoulides, D. N. Accelerating finite energy Airy beams. *Opt. Lett.* **32**, 979-981 (2007).
4. Zhang, P. et al. Nonparaxial Mathieu and Weber Accelerating Beams. Phys. Rev. Lett. **109**, 193901(2012).
5. Leutenegger, M., Rao, R., Leitgeb, R. A., Lasser, T. Fast focus field calculations. *Opt. Express* **14**, 11277-11291 (2008).
6. Gu, M. Advanced Optical Imaging Theory. Chapter **3**. (Springer, 1999).
7. Richards, B., Wolf, E. Electromagnetic Diffraction in Optical Systems. II. Structure of the Image Field in an Aplanatic System. *Proc. Royal Soc. A* **253**, 358-379 (1959).
8. Youngworth, K. S., Brown, T. G. Focusing of high numerical aperture cylindrical-vector beams. *Opt. Express* **7**, 77-87 (2000).
9. Wang, H., Shi, L., Lukyanchuk, B., Sheppard, C., Chong, C. T. Creation of a needle of longitudinally polarized light in vacuum using binary optics. *Nat. Photonics* **2**, 501-505 (2008).
10. Broky, J., Siviloglou, G. A., Dogariu, A., Christodoulides, D. N. Self-healing properties of optical Airy beams. *Opt. Express* **16**, 12880-12891 (2008).
11. Macdonald, R. P., Boothroyd, S. A., Okamoto, T., Chrostowski, J., Syrett, B. A. Interboard optical data distribution by Bessel beam shadowing. *Opt. Commun.* **122**, 169-177 (1996).
12. Zhou, C., Liu, L. Numerical study of Dammann array illuminators. *Appl. Opt.* **34**, 5961-5969 (1995).
13. Yu, J. et al. Three-dimensional Dammann array. *Appl. Opt.* **51**, 1619-1630 (2012).
14. Lin, H., Jia, B., Gu, M. Dynamic generation of Debye diffraction-limited multifocal arrays for direct laser printing nanofabrication. *Opt. Lett.* **36**, 406-408 (2011).
15. Ren, H., Lin, H., Li, X., Gu, M. Three-dimensional parallel recording with a Debye diffraction-limited and aberration-free volumetric multifocal array. *Opt. Lett.* **39**, 1621-1624 (2014).
16. Zhu, L., Yu, J., Zhang, D., Sun, M., Chen, J. Multifocal spot array generated by fractional Talbot effect phase-only modulation. *Opt. Express* **22**, 9798-9808 (2014).
17. Wang, X., Gong, L., Zhu, Z., Gu, B., Zhan, Q. Creation of identical multiple focal spots with three-dimensional arbitrary shifting. *Opt. Express* **25**, 17737-17745 (2017).
